# Tumor Microenvironment and Cell Fusion

**DOI:** 10.1155/2019/5013592

**Published:** 2019-07-11

**Authors:** Erhui Jiang, Tinglin Yan, Zhi Xu, Zhengjun Shang

**Affiliations:** ^1^The State Key Laboratory Breeding Base of Basic Science of Stomatology (Hubei-MOST) & Key Laboratory of Oral Biomedicine Ministry of Education, School & Hospital of Stomatology, Wuhan University, Wuhan, 430079, China; ^2^Tongji Medical College, Huazhong University of Science and Technology, Wuhan, 430030, China; ^3^Department of Oral and Maxillofacial-Head and Neck Oncology, School and Hospital of Stomatology, Wuhan University, Wuhan, 430079, China

## Abstract

Cell fusion is a highly regulated biological process that occurs under both physiological and pathological conditions. The cellular and extracellular environment is critical for the induction of the cell–cell fusion. Aberrant cell fusion is initiated during tumor progression. Tumor microenvironment is a complex dynamic system formed by the interaction between tumor cells and their surrounding cells. Cell–cell fusion mediates direct interaction between tumor cells and their surrounding cells and is associated with tumor initiation and progression. Various microenvironmental factors affect cell fusion in tumor microenvironment and generate hybrids that acquire genomes of both parental cells and exhibit novel characteristics, such as tumor stem cell-like properties, radioresistance, drug resistance, immune evasion, and enhanced migration and invasion abilities, which are closely related to the initiation, invasion, and metastasis of tumor. The phenotypic characteristics of hybrids are based on the phenotypes of parental cells, and the fusion of tumor cells with diverse types of microenvironmental fusogenic cells is concomitant with phenotypic heterogeneity. This review highlights the types of fusogenic cells in tumor microenvironment that can fuse with tumor cells and their specific significance and summarizes the various microenvironmental factors affecting tumor cell fusion. This review may be used as a reference to develop strategies for future research on tumor cell fusion and the exploration of cell fusion-based antitumor therapies.

## 1. Introduction

Cell fusion is a process that two or more cells become one by membrane fusion [1, 2]. Cell fusion is essential for fertilization, organ development, immune response, tissue repair, and regeneration under physiological conditions [3, 4]. In some pathological processes, such as infection and tumor progression, aberrant cell fusion occurs frequently [5]. As a major public health problem, malignant tumor is a leading cause of death worldwide [6, 7]. Thus, continued clinical and basic research is required to fight against tumor. Cell fusion participates in processes associated with tumor initiation and progression [8, 9]. The presumption that cell fusion plays critical roles in tumor appears since it was postulated by Otto Aichel in 1911 [10]. Tumor may originate from the accumulation of chromosomal abnormalities, such as the formation of aneuploidy or tetraploidy, which can cause the chromosomal damage and genetic instability and result in the malignant transformation of cells [11, 12], and cell fusion is an important way to generate polyploid cells [13, 14]. During tumor progression, cell fusion is involved in cancer stem cell formation [9, 15], high invasiveness acquisition [16], tumor microenvironment (TME) remodeling [17], epithelial–mesenchymal transition (EMT) [18], drug resistance [15], and tumor angiogenesis [19], which are closely related to the growth, invasion, and metastasis of tumor.

Previous researches on the relationship between cell fusion and tumor focused on the role of cell fusion in tumor stem cell origination and tumor metastasis. Tumor stem cells possess the ability to initiate a heterogeneous tumor and exhibit apparent changes in behavior associated with metastasis and recurrence [20, 21], which are the main causes of human tumor deaths [22, 23]. Tumor stem cells reportedly originate from primitive progenitor cells with cancerous mutations [24] or from normal stem cells with phenotypic changes or gene mutations [25]. An increasing number of studies indicate that the origination of tumor stem cells is closely related to cell fusion and that bone marrow-derived stem cells fuse with somatic cells or tumor cells to produce tumor stem cells [26–28]. In addition, tumor cells fuse with tumor cells or other somatic cells to generate hybrid cells that possess the genetic characteristics of both parental cells, leading to the apparent variation of the biological behaviors of tumor cells, such as decreased adhesion, enhanced invasion and migration, increased drug resistance, and enhanced proliferation and antiapoptotic ability [29–31]. Overall, cell fusion participates in the initiation of tumor stem cells and malignancy transformation of tumor cells, resulting in the recurrence and metastases of tumor.

TME is a complex dynamic system that includes tumor cells, the surrounding epithelial cells [32, 33], stromal cells composed of fibroblasts, immune cells, vascular endothelial cells, pericytes, adipocytes, bone marrow mesenchymal stromal cells, cytokines, vascular tissue, and the extracellular matrix [34]. Tumor cells interact with the surrounding stroma, exchange information, and dynamically remodel the microenvironment, thus creating a microenvironment conducive to events such as tumor angiogenesis, proliferation, invasion, metastasis, and therapeutic resistance [33, 35, 36]. Moreover, the factors influencing TME interaction may be secreting soluble factors, cytokines, microRNAs, or extracellular vesicles derived from tumor or stromal cells [37–39]. Microorganisms, such as bacteria and viruses, may also be involved in microenvironment remodeling [40–42]. Cell fusion is regulated by multiple genes and has an epigenetic tendency [2]. In general, cell fusion is strictly regulated by genes, and spontaneous fusion between different cells rarely occurs, but external stimuli or variation of the cellular and extracellular environment caused by aging, radiation exposure, inflammation, chemotherapeutic drugs, and tissue damage can influence cell fusion [43, 44]. As a malignant disease with genetic characteristics and biological behaviors that are different from those of normal tissues, tumor constructs a microenvironment apparently different from that in normal tissues, which would cause aberrant cell–cell fusion. For instance, TME is always deficient in oxygen and nutrients [45] and is usually characterized by relatively low pH [35, 46] and chronic inflammatory state [47]. Tumors use this relatively “harsh” environment to promote processes (e.g., cell fusion) that are related to their progression [8, 13, 48]. Actually, hypoxia caused by oxygen deficiency, and signal pathways activated by chronic inflammation, such as matrix metalloproteinase (MMP) and tumor necrosis factor (TNF) pathways, are involved in the regulation of cell fusion [18, 44, 49, 50]. These studies indicate that factors in TME are closely related to the fusion of tumor cells with tumor cells or other stromal cells.

The role of cell fusion in tumor initiation and progression has been fully discussed. As the hybrids derived from cell–cell fusion acquire genomes of both parental cells and exhibit phenotypic characteristics based on the phenotypes of parental cells, fusion of tumor cells with diverse types of fusogenic cells is concomitant with phenotypic heterogeneity. In this review, we will classify the fusogenic cells in TME that can fuse with tumor cells and discuss the specific significance of the fusion between tumor cells and other types of cells (Table 1). We will also summarize the various factors in the TME that affect tumor cell fusion (Table 2), expecting to provide new ideas for future research on tumor cell fusion and present strategies for antitumor therapies.

## 2. Tumor–Epithelial Cell Fusion

### 2.1. Fusion between Tumor Cells and Normal Epithelial Cells

As the saying goes, “lies down with dogs must rise up with flea,” the normal cells adjacent to the tumor cells would acquire the “evil” characteristics of tumor cells. During tumor progression, tumor cells can transmit malignancy to surrounding cells; one of the underlying mechanisms is cell–cell fusion [51]. Senescent normal human prostate epithelial cells can fuse with multiple types of tumor cells and generate tumor cells with increased heterogeneity and extended survivability to enhance their tumorigenicity [52]. It was also reported that breast cancer cells fused with breast epithelial cells to generate hybrid cell lines exhibiting properties of cancer stem/initiating cells and increased metastatic and drug resistant abilities [53]. In addition, only the hybrid cell lines but not parental cells respond to chemokine CCL21 stimulation with an increased migratory activity [54, 55]. Considering that the CCL21/ chemokine receptor 7 axis is associated with the metastasis of tumor cells to lymph nodes [56], this study suggests that cell fusion is a mechanism behind the origin of metastatic cancer cells in breast cancer. The proinflammatory cytokine TNF-*α* is involved in the tumor–epithelial cell fusion. TNF-*α* enhances the fusion of breast cancer cells and epithelial cells by upregulating the expression of MMP-9 in cancer or epithelial cells [50]. In our previous study, oral squamous carcinoma cells could fuse with human immortalized oral epithelial cells (HIOECs) spontaneously, and the fusion was enhanced by hypoxia-induced EMT of epithelial cells. Given that EMT is closely related to tumor invasiveness, a link possibly exists between cell fusion and tumor invasiveness [18].

Therefore, fusion between tumor cells and normal epithelial cells may imply two facts: one is that individual cells with accidental tumorigenic mutations spread their aberrant mutations by fusing with the surrounding epithelial cells, by which way mutations are amplified and accumulated and cause tumor initiation; the other is that tumor cells fuse with normal epithelial cells to reprogram their genomes and acquire novel malignant characteristics, such as cancer stem cell-like properties and increased metastatic ability, to promote tumor progression.

### 2.2. Fusion between Tumor Cells and Tumor Epithelial Cells

The phenomenon of tumor–tumor cell fusion is frequently observed and has been widely studied. Mi et al. [57] obtained stable tumor–tumor cell fusion hybrids in melanoma. They found that even if the cell size and the chromosome numbers of the hybrids were approximately twice those of the parents, the hybrids remained to have a stable genotype after several generations of cell proliferation* in vitro* and* in vivo*, and the tumor–tumor cell fusion hybrids acquired enhanced metastatic potential specific to the lungs. In view of the fact that tumor cells have undergone genetic mutations, tumor–tumor cell fusion means that the further accumulation of mutations would multiply the effects of genetic variation and cause the malignant transformation to contribute to the metastasis and recurrence of tumor.

Heterogeneity is a prominent feature of tumors; intratumor heterogeneity represents phenotypic diversity within a single tumor, which fosters tumor evolution [58]. Fusion of the heterogeneous cells may result in the combining of the heterogeneous characteristics. As early as in 1989, Miller et al. [59] obtained hybrids from spontaneous fusion of two sister tumor subpopulations that originated from a single mouse mammary tumor but with different drug resistance and metastasis capacities; and they found that the hybrids combined clinically aggressive features of both parental cells, suggesting that spontaneous fusion within the tumor lead to a more aggressive variant. After decades, Lu et al. [60] found that spontaneous fusion between bone- and lung-special metastatic sublines of the human breast cancer cell line acquired dual metastasis organotropisms* in vitro* and* in vivo*, which suggested that tumor–tumor cell fusion could act as an efficient mean of phenotypic evolution during tumor progression.

## 3. Tumor–Stromal Cell Fusion

### 3.1. Fusion between Tumor Cells and Bone Marrow-Derived Cells (BMDCs)

BMDCs fuse with somatic cells in different organ systems for tissue regeneration and as a response to injury [92–94]. Tumor tissue is comparable to a wound that would never heal, which recruits large numbers of BMDCs and provides the basis for the fusion of tumor cells and BMDCs. Fusogenic BMDCs are reported to be involved in carcinogenesis, tumor heterogeneity, and acquisition of stem-like, and metastatic properties [95]. Researchers transplanted bone marrow from female mice into male mice to generate sex-mismatched chimeric mice and induced gastric cancer in the chimeric mice. They found that BMDCs participated in the renewal of gastric mucosa in both precancerous lesions and adenocarcinoma [61]. Spontaneous fusion between breast cancer and BMDCs generates hybrids that are considered as a source of tumor heterogeneity in invasive breast cancer [62]. BMDCs fuse with low metastatic-human liver cancer cells and impart characteristics of malignant cells to the cancer (hybrid) cells. The hybrids exhibit enhanced EMT and promote the abilities of invasion and migration [64]. During tumor progression, hematopoietic cells are recruited to the tumor site to fuse with tumor cells and impart hematopoietic characteristics to tumor cells. Ramakrishnan et al. [63] reported that hemato-epithelial cancer cells existed in ovarian cancers. The hemato-epithelial cancer cells express hematopoietic markers and possess stem cell phenotypes and are found to originate from the fusion of ovarian cancer cells with recruited BMDCs. Hemato-epithelial cancer cells (hybrids) maintain epithelial phenotype and gain promigratory property by activating the CXCR4/CXCL12 axis through fusion. In mouse prostate tissues, CD45+ BMDCs are recruited to fuse with prostate cells to promote tumor growth in vivo [29].

In 2013, researchers from Yale found direct evidence supporting that fusion between donor BMSCs and patient cells as the initiator of metastasis in a patient who received allogeneic bone marrow transplants and later developed melanoma brain metastasis. They detected all the alleles of the donor and patient in the metastatic tumor cells. And this was the first case of BMSC-tumor cell hybridization in human cancer metastasis [96]. Later in 2017, they found a second case in a patient with melanoma lymph node metastasis [97]. Their founding suggests that fusion between tumor and BMDCs plays a critical role for melanoma and other solid tumor metastases.

### 3.2. Fusion between Tumor Cells and Mesenchymal Stem/Multipotent Stromal Cells (MSCs)

Mesenchymal stem/multipotent stromal cells are abound in TME and play critical roles during tumor progression. MSCs can fuse with tumor cells and impart their stemness characteristics to tumor cells. Wang et al. [68] used laser-induced single-cell fusion to fuse hepatocellular cancer cells and human embryonic stem cells and generated tumor initiating-like hybrids with stemness and cancer cell-like characteristics. They found that the hybrids exhibited increased drug resistance and tumorigenesis. Besides, as the characteristics of the hybrids are associated with the recurrent cancer stem cells (rCSCs), which cause therapy resistance and highly malignant recurrences, researchers speculate that cell fusion may be the origin of rCSCs [65]. During these courses, the TNF-*α* signaling pathway is involved in the fusion between cancer cells and MSCs to generate tumor initiating-like hybrids [66]. Fusion of MSCs with tumor cells is also related to the malignance of tumor cells. Noubissi et al. [67] found that hypoxic conditions stimulated the fusion between MSCs and breast tumor cells by inducing apoptosis, generating hybrids with an enhanced migratory capacity. Interestingly, the fusion was enhanced only between nonmetastatic breast cancer cells (T47Ds and MCF7s) and MSCs but not metastatic cells (MDA-MB-231s or MCF10As), indicating that hypoxia-induced fusion was critical for the malignant transformation of the nonmetastatic cancer cells, which is not necessary or advantageous for the already metastatic cells.

However, fusion between MSCs and tumor cells exerts tumor-inhibiting effects. For instance, MSCs fused with lung cancer cells (H441) induce a reprogramming of the cancer cell transcriptome. Then, the fusion-induced transcriptome reprograming complements the tumorigenic defects of cancer cells by restoring the function of p21 and upregulating the tumor suppressor FOXF1; thus, the hybrids exhibit a growth-suppressed state [70]. Besides, Ke et al. (108) found that the apoptotic signal was much stronger and growth was suppressed in hybrid cells of human embryonic stem cells and ovarian cancer cells.

Altogether, the hybrids derived from fusion between MSCs and tumor cells show diversification, which may be attributed to the high plasticity and multipotency of both MSCs and tumor cells. Through cell–cell fusion, tumor cells can acquire the stemness and migratory capacity of MSCs to promote tumor progression; MSCs, however, can complement the tumorigenic defects of tumor cells by reprogramming their genomes. Further explorations are needed to clarify whether characteristics the hybrids appear to exhibit depend on the location and type of the genetic mutation.

### 3.3. Fusion between Tumor Cells and Immune Cells

Through fusion, fusogenic immune cells transfer distinct cellular capabilities to tumor cells and confer migratory (metastatic) and immune evasion capabilities. Cell fusion between tumor cells and macrophage generates hybrids that retain the characteristics of both parental cells and acquire physical behavior attributed to migratory macrophages, including navigation of circulation and immune evasion [74]. Researchers from Yale were the first to report that fusion between solid tumors like melanoma and macrophages enhances tumor metastasis in 1998 [71]. They found that fusion between weakly metastatic mouse melanoma cells and human or mouse macrophages generated hybrids exhibiting enhanced metastatic abilities, and they further revealed that altered N-linked glycosylation was an underlying mechanism for regulation of the metastasis in the melanoma-macrophage hybrids [72, 73, 95].

Acute myeloid leukemia (AML) cells spontaneously fuse with murine host cells, including macrophages, dendritic, and endothelial cells, in a mouse AML model. The hybrids from the mouse model give rise to leukemia with 100% penetrance when reinjected in a secondary recipient, suggesting that cell–cell fusion is a mechanism of gene transfer for cancer dissemination [98]. Recently, Gast et al. [79] reported that fusion between cancer cells and macrophage contributed to the heterogeneity and increased metastatic behavior of the tumor* in vitro* and* in vivo*. They found that the fusion hybrids possessed both the hematopoietic and epithelial properties and could also be detected in the peripheral blood of human cancer patients. This population of unique fusion hybrids was associated with tumor stage and predicted overall survival. Fusion between tumor and macrophage also generates hybrids with radioresistant ability. Lindström et al. [75] reported that M2-macrophages fused with breast cancer cells and induced the radioresistance of the hybrids by enhancing their DNA-repair capacity. The metastatic properties of defined target organ specificity may be attributed to cell fusion between tumor cells and normal cells of the lymphoreticular system. Target organ specificity is based on the cell surface marker encoded by the genome derived from normal parental cells [80]. Cancer cells fuse with macrophage and generate hybrids with macrophage phenotypic characteristics that express the macrophage-specific marker CD163 [76]. The hybrids expressing CD163 acquire radioresistance and exhibit improved survival and colony forming ability. CD163 expression implies advanced stages and poor prognosis in breast cancer [77]. Researchers suggest that the acquisition of new malignant characteristics of hybrid cells may be attributed to the fact that tumor-macrophage fusion acquires the properties of cancer stem cells [78]. Moreover, fusion between circulating immune cells and tumor epithelium cells occurs during tumorigenesis [74]. Furthermore, cell–cell fusion might well explains the metabolic phenotype of tumor cells. Malignant tumor cells tend to undergo aerobic glycolysis to provide substrates for protein and nucleic acid synthesis while meeting their energy requirements [99]. Fusion between cancer cells and macrophage generates hybrids that demonstrate a high level of constitutive autophagy, which is the characteristic of macrophage under hypoxia and nutrient deprivation as part of the Warburg effect. The fusion-derived macrophage characteristics allow tumor cells to survive and proliferate under adverse microenvironment of hypoxia and deprivation [100].

During antitumor immune responses, antigen-presenting cells (APCs) are responsible for presenting tumor-associated antigens (TAAs) to activate T cells. Dendritic cells are powerful APCs for the induction of antitumor immunity [101]. Researchers administer the fusion between dendritic cells and whole tumor cells by chemical, physical, or viral means and generate hybrids that display both known and unidentified TAAs originally expressed by whole tumor cells. Then, the dendritic-tumor hybrids process multiple TAAs endogenously and present antigenic peptides to both major histocompatibility complex classes I and II molecules to activate both CD4^+^ and CD8^+^ T cells, thus activating the antitumor immunity [81, 82, 102, 103]. Dendritic–tumor cell fusion provides strategies to produce dendritic-tumor cell fusion-based tumor vaccines to display immunotherapy for tumors.

### 3.4. Fusion between Tumor Cells and Stromal Fibroblasts

Fibroblasts are the most abundant stromal cells in TME. Fibroblasts in TME are activated by tumor cells to form cancer associated fibroblasts (CAFs), which play critical roles in processes associated with tumor progression [104]. CAFs interact with tumor cells and secrete cytokines, inflammatory cytokines, and extracellular vesicles to the microenvironment and directly supply metabolites to tumor cells to support their survival and proliferation [105]. Recently, tumor–fibroblast cell fusion has been considered to be a part of the tumor–stroma interaction. CAFs fuse with tumor cells and generate hybrids in coculture model. Most of the hybrids remain growth-arrested and eventually perished, but some of the hybrids survive and gain a strong clonogenic capacity. The survived hybrids experience genomic alterations and acquire novel malignant characteristics, which are the potential cause of tumor invasion and recurrence. Fusion of fusogenic CAFs with prostate cancer cells generates a derivative cancer cell population with increased malignancy and androgen-independent phenotype [83]. Fusion of tumor cells and stromal fibroblasts also transmits malignancy horizontally [51]. Searles et al. [84] used a Cre-loxP system to observe fusion between melanoma (B16) and primary fibroblasts or macrophages. They found that tumor cells exchanged functional consequences of DNA with noncancer cells to generate hybrids with enhanced clonal diversity and chemoresistance. Their results highlighted the efficiency of cell fusion as a mechanism by which cancer cells attained aneuploidy numbers of chromosomes and gained the phenotypic heterogeneity to survive in a given selective pressure. Notably, hybrids cannot be distinguished from parental stromal cells based on their morphology and immunophenotype, rendering the tumor cells undetectable by routine histopathological assessments and indicating the existence of recurrent tumor-initiating cells in melanoma [85, 86].

### 3.5. Fusion between Tumor Cells and Vascular Endothelial Cells

**Figure 1:**
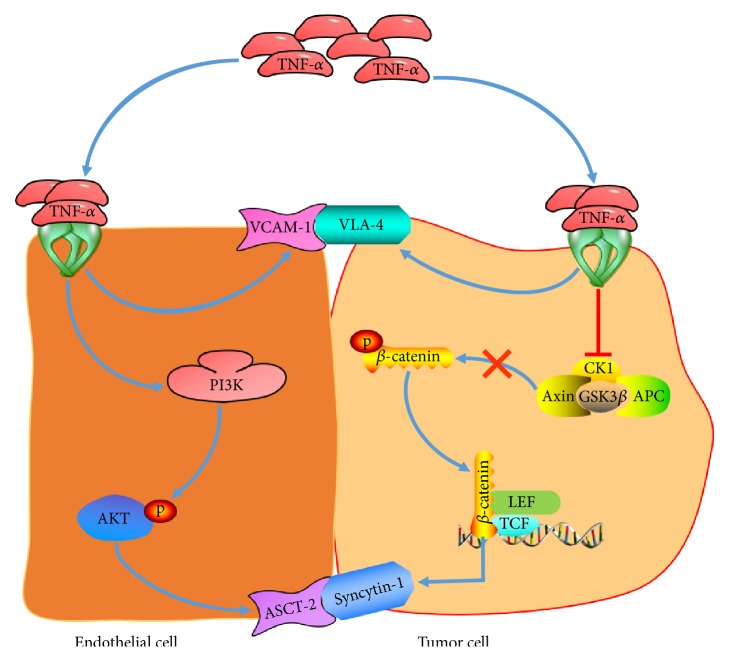
Schematic summarizing fusion between tumor cells and endothelial cells under TNF-*α*-induced inflammatory environment in our previous studies. TNF-*α* upregulated the expression of VCAM-1 and ASCT-2 on vascular endothelium and upregulated the expression of VLA-4 and syncytin-1 on cancer cells, respectively, to promote tumor–endothelial cell fusion.

Vascular endothelial cells are abundant stromal cells in TME. During tumor progression, interaction between cancer cells and endothelial cells is critical for both tumor angiogenesis and metastatic dissemination [106]. Spontaneous fusion between cancer cells and endothelial cells occurs in various tumors* in vitro* and* in vivo*, suggesting that fusion is an important type of tumor–endothelial cell interaction and might strongly affect the biological behavior of tumors [107]. In our studies, we elucidated the underlying mechanism involved in oral cancer–endothelial cell fusion and found that oral cancer cells could spontaneously fuse with endothelial cells* in vitro* and in* vivo*. After the fusion, the hybrids expressed markers of both parental cells and acquired drug resistance and enhanced survival potential [30]. Then, we explored whether the oral cancer–endothelial cell fusion was promoted in the inflammatory TME. We modeled the inflammatory environment with the proinflammatory cytokine TNF-*α* (Figure 1). Afterward, we found that the fusion between oral cancer cells and vascular endothelial cells was apparently enhanced in TNF-*α*-induced inflammatory environment. During these process, TNF-*α* upregulated the expression of vascular cell adhesion molecule-1 (VCAM-1) and amino acid transporter type 2 (ASCT-2) on vascular endothelium and upregulated the expression of very late activation antigen 4 (VLA-4) and syncytin-1 on cancer cells, respectively [87, 88], which played critical roles in promoting oral cancer–endothelial cell fusion. Cell fusion-mediated tumor–endothelial cell interaction may promote tumor angiogenesis and allow disseminated tumor cells to pass through the endothelium into the circulatory system. Our works were the first to study the influence of the inflammatory factor on cancer–endothelial cell fusion and explore the correlation among inflammation, cell fusion, and cancer, providing important insights into the underlying mechanisms of cancer–endothelial cell direct interaction in TME.

## 4. Tumor Microenvironmental Factors Affecting Cell Fusion

### 4.1. Microenvironmental Microorganism and Tumor Cell Fusion

Infection of virus or bacterium is a primary cause of several cancers [108], and cell fusion has been defined as a link between infections and cancer [109]. Microenvironmental oncogenic viruses, such as human papillomavirus (HPV) [110, 111], Epstein-Barr virus [112], hepatitis B virus [113], hepatitis C virus [114], Kaposi sarcoma herpesvirus [115], and human T-cell lymphotropic virus type 1 [116], can induce cell fusion [117]. The fact that tetraploid cells are more frequently observed in virus-positive than in virus-negative lesions in some virus-related tumors [118] implies that some links exist between virus-mediated cell fusion and tumor. Further study shows that fusogenic viruses, even the traditionally nonneoplastic viruses, cause massive chromosomal instability by inducing cell fusion and initiate tumorigenesis or enhance the malignant properties of tumor [89].

Considering that HPV infection is closely related to cervical cancer progression, Peng et al. [90] analyzed the tetraploid cells and the HPV infection state in precancerous cervical lesions and found that the formation of tetraploid cells by cell fusion was obviously associated with HPV infection. They suggested that HPV-induced cell fusion was an important initiating event in the early stage of HPV-associated cervical cancer.

### 4.2. Hypoxia and Tumor Cell Fusion

Hypoxia is a common condition of TME in solid tumors and is related to poor prognosis. During tumor progression, hypoxia is associated with processes, such as EMT and apoptosis, that increase tumor invasion and metastasis [119]. Hypoxia is a strong inducer of cell fusion in TME [91]. Noubissi et al. [67] reported that hypoxia-induced-apoptosis stimulated fusion between MSCs and breast tumor cells and enabled the metastatic capacity of nonmetastatic breast cancer cells. In our study, hypoxia promotes the spontaneous cell fusion between oral squamous carcinoma cells and oral epithelial cells by enhancing the EMT of epithelial cells [18].

### 4.3. TNF-*α* Signaling Pathway and Tumor Cell Fusion

Chronic inflammation is a critical characteristic in TME. As a proinflammatory cytokine, TNF-*α* has a wide range of roles during the development of tumors. TNF-*α* is synthesized and secreted by malignant tumor cells and is abound in TME. It can trigger inflammatory cell infiltration, activate pathways associated with cancer cell survival and proliferation, and promote angiogenesis and tumor cell migration and invasion [120]. TNF-*α* is an important factor mediating cell–cell fusion, especially under hypoxic conditions in TME [91]. Melzer et al. [66] found that TNF-*α* mediated fusion between breast epithelial cells and MSCs by activating TNF receptor-induced cell death pathways or additional NF-*κ*B signaling and contributed to the formation of tumor stem-like cells. In our previous studies (Figure 1), we found that TNF-*α* increased the spontaneous fusion between oral cancer cells and vascular endothelial cells. TNF-*α* upregulated the expression of VCAM-1 in vascular endothelium, which is a ligand of very late activation antigen 4 (VLA-4) on cancer cells, and finally enhanced the adhesion and fusion between oral cancer cells and vascular endothelial cells through the VCAM-1/VLA-4 pathway [87]. We also found that TNF-*α* upregulated the expression of syncytin-1 in oral cancer cells and its receptor ASCT-2 in vascular endothelial cells, respectively. Syncytin-1 is an important fusogen that mediates cell–cell fusion [1], and it was upregulated through the Wnt/*β*-catenin pathway; thus, we concluded that TNF-*α* promoted fusion between oral cancer cells and vascular endothelial cells via the Wnt/*β*-catenin-dependent upregulation of syncytin-1 [88]. Recently, Weiler et al. [50] found that MMP9 was also involved in the TNF-*α*-mediated fusion of breast cancer cells and epithelial cells.

Altogether, tumor hybrid cells generated in chronically inflamed TME demonstrate improved proliferation and invasiveness and produce therapy-resistant cancer hybrid stem cells [121] to promote tumor progression.

### 4.4. Other Factors

Aside from microorganisms, hypoxia, and inflammation, other factors associated with tissue injury, cell proliferation, and intercellular communication may also be involved in cell fusion [44]. As mediators of intercellular communication, extracellular vesicles (EVs), including microvesicles and exosomes, transport biological cargoes, including proteins, nucleic acids, and lipids, to recipient cells and have a wide range of biological functions [122, 123]. EVs may transport cargoes related to cell fusion. A previous study reported that tumor-derived exosomes did not have positive effect on promoting cell fusion, but even impaired cell fusion [91]. However, researchers found that EVs carried fibronectin that triggered a proinflammatory situation and syncytin that was involved in membrane fusion and were abound in Wnt/*β*-catenin-related molecules associated with cell fusion; thus, EVs have great potential to promote cell–cell fusion [124]. Thus, the association between EVs-mediated intracellular communication and cell–cell fusion in TME still needs further research. Some biochemical and biophysical factors were also proved to regulate cell–cell fusion, such as nanotopography and stiffness [125, 126], which provided more possibilities for artificial intervention of cell fusion in TME.

## 5. Conclusion

**Figure 2:**
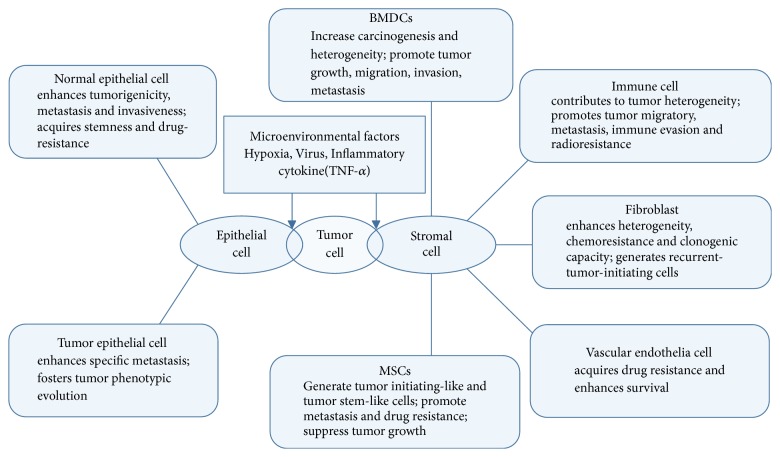
Summary of the types of fusogenic cells in tumor microenvironment that can fuse with tumor cells and their specific significance in tumor progression and the various microenvironmental factors affecting tumor.

Mutations and accumulation of genetic aberration are thought to be the principal pathways by which cells undergo malignant transformation. Cell fusion is an efficient process of rapid phenotypic and functional evolution that produces cells with new properties at a much higher rate than random mutagenesis. Thus, cell fusion is beneficial to the evolving cancer cell population by gaining novel properties to help tumor cells survive in a given selective pressure. Through fusion with diverse types of cells in TME, tumor cells reprogram their genomes and exhibit malignant characteristics associated with tumor progression (Figure 2): (1) generate tumor-initiating cells and tumor stem-like cells and provide seeds for tumor initiation and recurrence; (2) enhance capabilities of migration, invasion, and angiogenesis and promote tumor cells to leave the primary tumor and invade the circulatory system; (3) acquire capabilities of radioresistance, drug resistance, and immune evasion and allow tumor cells to survive in the circulatory system; and (4) contribute to specific metastasis, enhance tumor heterogeneity and clonogenic capacity, and allow the colonization and proliferation of tumor cells. Thus, we conclude that cell fusion participates in steps throughout tumor progression and that investigating the effects of fusion between tumor cells and different microenvironmental cells, exploring the factors affecting cell–cell fusion in tumor microenvironment and figuring out the underlying mechanisms are of great significance.

## 6. Prospects

Although cell–cell fusion in TME and the relationship between cell fusion and tumor progression have been studied for several decades, the mechanism by which the fusion of tumor cells drives the biology of cancer remains unclear to date and strategies for cell–cell fusion-based antitumor therapy are limited. For instance, some previous studies did not strictly distinguish cell entosis and cell fusion, both of which could generate hybrids. Moreover, microenvironmental factors affecting cell–cell fusion did not attract enough attention from researchers. As important mediators of intracellular communication, EVs and their roles in tumor cell fusion were less studied, and whether the fusogenic cells in TME undergo metabolic reprogramming remained unclear. Besides, the low incidence of spontaneous cell fusion and the constant reprogramming of genome in fused hybrids make it difficult to monitor cell fusion during tumor progression by the traditional methods [127]. And the studies of fusion in tumor were limited to* in vitro* and animal studies. Recently, a new technology, single-cell RNA sequencing (scRNA-seq), emerges as a revolutionary tool to detect the genome of a single cell at a microscopic resolution and decipher gene expression dynamics [128], which is an excellent technology to study tumor heterogeneity. And scRNA-seq can be used to detect an incidental fusion event in tumor and monitor the dynamics of the fused hybrids during tumor progression.

In this review, we summarize the diverse types of fusogenic cells and the possible fusion-associated factors in TME. This paper tries to draw attention to cell fusion and TME and provides new ideas for future research on tumor cell fusion and presents potential cell fusion-based strategies for antitumor therapies.

## Figures and Tables

**Table 1:** Different cell types fused with tumor cell.

Cancer	Cells fused with cancer cells	Significance	Signal pathway involved	References
Breast cancer	Breast epithelial cells	Origin of stem cell and metastatic cancer (hybrid) cells; drug resistance	CCL21/CCR7; TNF-*α*/MMP-9	[50, 53–55]
Prostate cancer	Prostate epithelial cells	Enhance the tumorigenicity of low-tumorigenic prostate cancer	p16 and hTERT	[52]
Oral cancer	Oral epithelial cells	Indicate a link between cell fusion and tumor invasiveness	Hypoxia-induced EMT	[18]
Melanoma	Melanoma	Enhance specific metastasis to the lungs	-	[57]
Breast cancer	Heterogeneous breast cancer cell	Foster tumor phenotypic evolution	-	[59, 60]
Gastric epithelial	BMDCs	Increase carcinogenesis	-	[61]
Breast cancer	BMDCs	Source of tumor heterogeneity	-	[62]
Ovarian cancer	BMDCs	Contribute to stem cell and migratory phenotypes	CXCR4/CXCL12	[63]
Prostate cancer	BMDCs	Promote tumor growth	-	[29]
Liver cancer	BMDCs	Promote invasion and migration	EMT	[64]
				
Breast cancer	MSCs	Promote metastasis and drug resistance; generate (recurrence) cancer stem cells	Hypoxia-induced apoptosis; TNF-*α* signaling pathway	[65–67]
Hepatocellular cancer	Embryonic stem cells	Generate tumor initiating-like cells	-	[68]
Ovarian cancer	Embryonic stem cells	Induce apoptosis and suppress the growth of tumor	p53 and PTEN	[69]
Lung cancer	MSCs	Reprogram cancer cell transcriptome and suppress tumor growth.	FOXF1/ p21	[70]
Melanoma	Macrophage	Enhances tumor metastasis	N-linked glycosylation	[71–73]
Intestinal cancer	Macrophage	Acquire capabilities of migratory and immune evasion	-	[74]
Breast cancer	Macrophage	Generate metastatic hybrids with cancer stem cell properties; induce the radioresistance	Enhance DNA-repair capacity	[75–78]
Intestinal cancer / melanoma	Macrophage	Contribute to tumor heterogeneity; increase metastatic behavior	-	[79]
Mouse plasmacytoma	B lymphocyte	Acquisition of metastatic properties	-	[80]
Whole tumor	Dendritic cells	Activate the antitumor immunity	Gain tumor-associated antigens	[81, 82]
Prostate cancer	Fibroblast	Acquire strong clonogenic capacity and androgen-independent phenotype	-	[83]
Melanoma	Fibroblasts/macrophage	Enhance heterogeneity and chemoresistance; generate recurrent-tumor-initiating cells	DNA exchange	[84–86]
Oral cancer	Endothelial cells	Acquire drug resistance and enhanced survival	VCAM-1/VLA-4; Wnt/*β*-catenin/ syncytin-1	[30, 87, 88]

**Table 2:** Tumor microenvironment factors affecting cell fusion.

Factor	Role	Significance	References
Virus	Enhance cell fusion	Cause chromosomal instability and initiate HPV-associated cancer	[89, 90]
Hypoxia	Promote cell fusion	Promote tumor invasiveness and metastasis	[18, 67, 91]
TNF-*α*	Promote cell adhesion and fusion	Generate tumor stem-like cells; acquire drug resistance and enhance survival	[50, 66, 87, 88, 91]
